# Incidence of vitamin D deficiency in adolescent idiopathic scoliosis: a meta-analysis

**DOI:** 10.3389/fendo.2023.1250118

**Published:** 2023-10-11

**Authors:** Clara Inés Llopis-Ibor, Gonzalo Mariscal, Jose Enrique de la Rubia Ortí, Carlos Barrios

**Affiliations:** ^1^ Institute for Research on Musculoskeletal Disorders, Valencia Catholic University, Valencia, Spain; ^2^ Department of Basic Medical Sciences, Catholic University of Valencia, Valencia, Spain

**Keywords:** vitamin D deficiency, vitamin D insufficiency, adolescent idiopathic scoliosis, 25hydroxy vitamin D, meta-analysis

## Abstract

**Introduction:**

This study aimed to investigate the incidence of vitamin D deficiency in patients with adolescent idiopathic scoliosis through a meta-analysis and to analyze trends and risk factors.

**Methods:**

Potentially relevant studies were searched using the terms “Vitamin D AND scoliosis.” Data on the incidence and risk factors, such as race, curve magnitude, and sex, were extracted from the selected studies. Review Manager 5.4 software was used for the data analysis. Six studies with a total of 1, 428 patients met the inclusion criteria.

**Results:**

The incidence of vitamin D insufficiency in patients with idiopathic scoliosis was 36.19% (95% CI [21.93 to 50.46]. In contrast, the incidence of vitamin D deficiency was 41.43% (95% CI [16.62 66.23]. Vitamin D levels were compared between Caucasian and African patients and it was concluded that Caucasian patients had a lower risk of vitamin D deficiency [RR 0.15, 95% CI (0.03 to 0.82; P = 0.03]. There was also an association between patients with idiopathic scoliosis and lower vitamin D -5.58, 95% CI [-7.10, -4.06]. Finally, no significant differences were observed in terms of curve magnitude assessed with the Cobb angle mean difference (MD) 4.45, 95% CI [-0.55, 9.44], or sex with lower-than-normal levels of vitamin D OR 0.96, 95% CI [0.58 to 1.60].

**Discussion:**

The incidence rates of vitamin D insufficiency and deficiency in patients with adolescent idiopathic scoliosis were 36.19% and 41.43%, respectively. The Caucasian race was associated with a lower risk of vitamin D deficiency compared to the African race. Vitamin D deficiency was not related to curve magnitude or sex.

## 1 Introduction

Adolescent idiopathic scoliosis (AIS) is a common orthopedic disorder that affects millions of children worldwide. Despite extensive research, the underlying cause of AIS remains unknown (1). Recent studies have suggested that various factors may contribute to the pathogenesis of AIS, including levels of leptin and ghrelin (2); estradiol levels (3); melatonin and calmodulin levels; growth hormone levels (4); and parathyroid hormone (PTH), serum calcium and vitamin D levels (5).

Vitamin D deficiency has been identified as a potential contributor to AIS pathogenesis. Regulation of calcium-phosphate metabolism in the skeletal system appears to be an important factor in the development of AIS (6). Vitamin D deficiency may influence the pathogenesis of AIS through its effects on bone metabolism as it interacts with estrogen, melatonin, leptin, bone mineral content, and bone mineral density (7).

Vitamin K2 has also been investigated for its role in regulating bone metabolism. It facilitates the deposition of calcium in bones by carboxylating osteocalcin, a protein produced by osteoblasts, during bone formation. Osteocalcin synthesis is induced by 1,25-dihydroxyvitamin D3. Vitamin D deficiency reduces the carboxylation of osteocalcin, resulting in reduced bone mineral density and osteopenia, which could make the vertebrae more susceptible to deformity and the development of AIS through the progression of the curve (6, 8). Reduced vertebral bone mineral density has been associated with a higher risk of curve progression and larger Cobb angles, as sustained compressive loading can cause progressive vertebral slippage in osteopenic vertebrae and vertebral wedging (7). The sex of patients could also influence AIS, given that differences between men and women in vitamin D metabolism have been evidenced (9); specifically, lower levels of vitamin D have been observed in women, which seems to explain the differences between sexes in terms of body composition with greater fat accumulation in women (10). Differences in vitamin D metabolism based on race are also well established, with black individuals tending to have lower cutaneous synthesis of vitamin D in response to solar radiation than individuals of other races (11).

Given the potential role of vitamin D deficiency in AIS pathogenesis, it is important to understand its incidence and possible associations with other risk factors. Therefore, the primary objective of this study was to analyze the incidence of vitamin D deficiency in patients with AIS to determine its prevalence and possible associations with other risk factors such as race, curve magnitude, and sex. This information may contribute to the development of preventive and therapeutic strategies against AIS.

## 2 Materials and methods

### 2.1 Eligibility criteria

This study followed a written protocol that included review questions, search strategy, inclusion/exclusion criteria, and risk of bias assessment. Meta-analysis was conducted in accordance with the PRISMA (Preferred Reporting Items for Systematic Reviews and Meta-Analyses) guidelines (12). The inclusion criteria followed the PICOS standard: patients with adolescent idiopathic scoliosis; this study is a meta-analysis of incidences and did not involve any interventions; however, the intervention group was composed of patients with vitamin D deficiency, the comparison was made with a control group of patients with adequate vitamin D levels; the main outcomes were to assess the incidence and risk factors, as well as trends related to vitamin D deficiency; cohort studies (prospective and retrospective); and clinical series. Exclusion criteria were neurological or muscular disorders, congenital malformations or genetic syndromes, non-idiopathic scoliosis, case reports, and studies with incomplete data, duplicate data, or non-shared variables.

### 2.2 Information sources

A systematic literature search was conducted using PubMed, EMBASE, Scopus, and Cochrane Collaboration Library databases. The search equation used to identify MeSH terms was “Vitamin D AND Scoliosis” (**Supplementary File 1**).

### 2.3 Search methods for identification of studies

Two reviewers independently agreed on the selection of eligible studies and reached a consensus regarding which studies to include. The records of the studies and their **Supplementary Material** were analyzed. Additionally, the studies of interest that appeared in the references of the studies included in the initial search were evaluated.

### 2.4 Data extraction and data items

Two authors independently reviewed studies on data extraction. If consensus could not be reached, a third author of the review was asked to complete the data extraction form. Additionally, the opinions of experts were consulted to evaluate the variables that would be of the greatest interest. Demographic values such as sex, age, race, calcium level, Cobb angle, season, phosphorus level, and BMI were collected based on the data provided by each study. The main variable was the incidence of vitamin D deficiency (<20 ng/mL) and insufficiency (levels–20-29 ng/mL) in adolescent idiopathic scoliosis patients. In addition, the influence of vitamin D deficiency was analyzed according to sex, curve magnitude, and race.

### 2.5 Assessment of risk of bias in included studies

The quality of the included studies was independently assessed by two authors using the Methodological Index for Non-Randomized Studies (MINORS) criteria (13) (**Table 1**). The maximum score was 24 for the comparative studies and 16 for the non-comparative studies. For non-comparative studies, scores of 0–4, 5–7, 8–12 corresponded to very low, low, fair, and ≥ 13corresponded to high quality, respectively. For comparative studies, scores of 0–6, 7–10, 11–15, and ≥ 16 corresponded to very low, low, fair, and high quality, respectively.

**Table 1:** Baseline characteristics of the six included studies.

Study	Region	Study type	Total patients	Age	No. of females	No. of males	Deficiency levels	MINORS
**Herdea et al., 2020** (14)	Romania	Cohort study	101	11-14	76	25	Insufficiency20-29ng/mL, deficiency<20ng/mL	13
**Balioglu et al., 2017** (5)	Turkey	Case-control study	618	10-22	344	274	Insufficiency 20-29ng/mL, deficiency <20ng/mL	12
**Hampton et al., 2022** (15)	UK	Cohort study	201	13-18	177	24	Suboptimal 50-75nmol/L, deficiency <50nmol/L	13
**Mayes et al., 2017** (16)	USA	Case series	217	10-17	145	72	Insufficiency 20-32ng/mL, deficiency <20ng/mL	10
**Batista et al., 2014** (6)	Brazil	Case-control study	115	4-47	99	16	–	12
**Beling et al., 2020** (17)	USA	Cross-sectional study	176	10-25	144	32	Insufficiency 20-29ng/mL, deficiency <20ng/mL	11

### 2.6 Assessment of the results

The incidence of vitamin D deficiency was calculated as the total number of patients with scoliosis and vitamin D deficiency divided by the total number of patients with scoliosis. In studies where the standard error (SE) was not reported, it was calculated from the prevalence using the following formula: SE = √p (1–p)/n & 95% CI = p Å} 1.96 X SE, where p = prevalence. Pooled incidences with 95% confidence intervals (CI) were calculated using a random-effects model (18). Meta-analysis was conducted using the Review Manager 5.4 software package provided by the Cochrane Collaboration. Odds ratios and risk ratios with 95% confidence intervals (CI) were calculated for dichotomous variables. The mean difference (MD) and 95% CI were calculated for continuous variables. Heterogeneity was assessed using both the χ2 and I2 tests. I2 values ranged from 0 to 100%, with values of 25%, 50%, and 75% indicating low, moderate, and high heterogeneity, respectively. A fixed-effects model was adopted if there was no statistical evidence of heterogeneity and a random-effects model was adopted if significant heterogeneity was observed. If the variables of interest could not be compared, narrative review was conducted.

### 2.7 Additional analyses

The possibility of publication bias was assessed using a funnel plot (Review Manager 5.4 software package).

## 3 Results

### 3.1 Study selection

**Figure 1:**
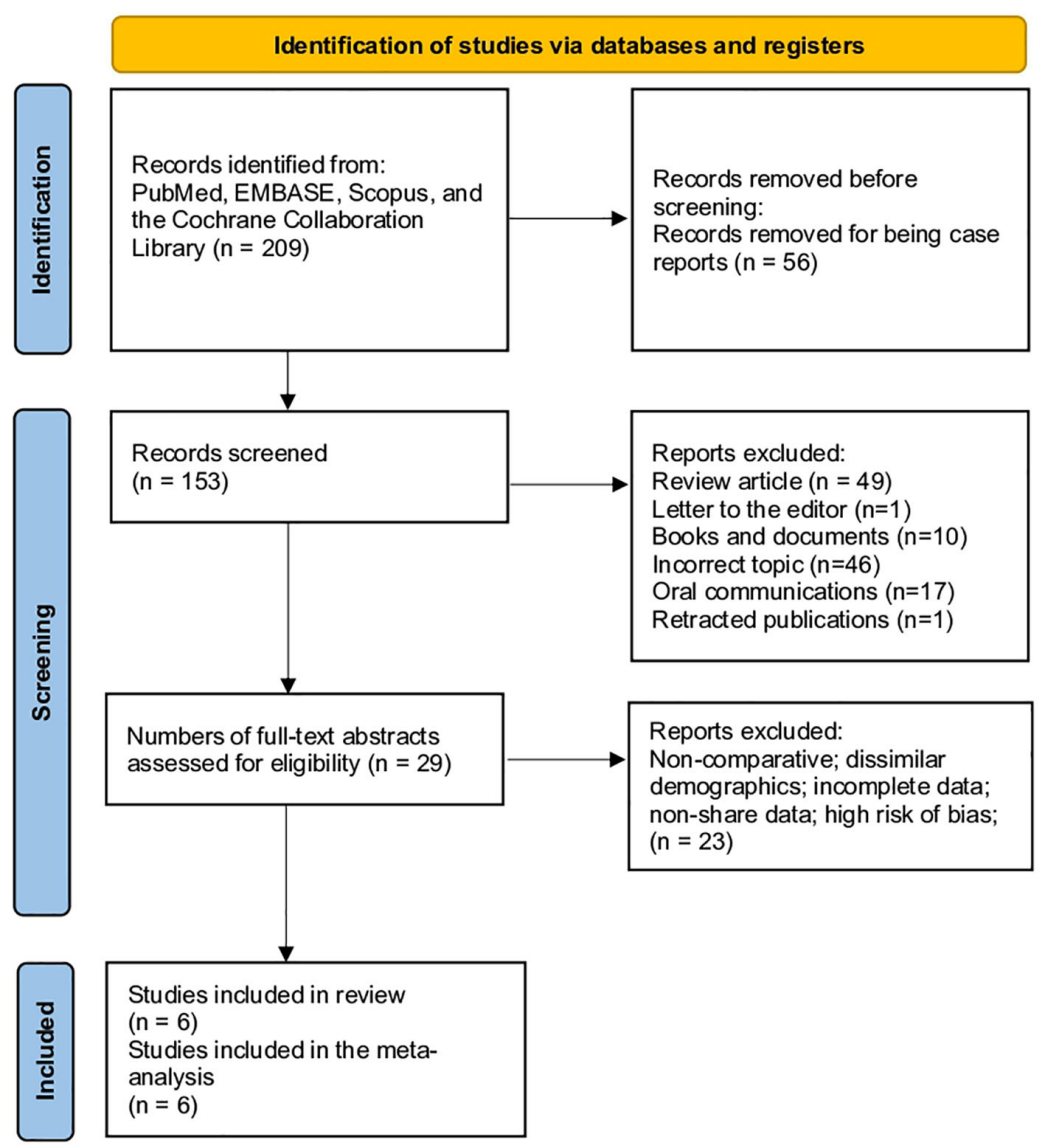
Flowchart of the search according to PRISMA criteria.

As shown in **Figure 1**, six articles were selected from a total of 209 articles initially searched. The selection process involved the exclusion of 56 articles that were found to be duplicates in both search engines. Subsequently, 124 articles were excluded based on the exclusion criteria and articles that were not relevant to the topic at hand. A total of 29 studies were analyzed, and 23 articles were excluded because they did not report vitamin D levels in patients. Ultimately, six articles were included in the meta-analysis (**Figure 1**).

### 3.2 Study characteristics

**Table 1** shows the demographic characteristics of the six selected studies. A total of 1428 patients were included in the study. Two cohort studies, two case-control studies, one cross-sectional study, and one case series were included. The percentage of women was 985/1428 (69.0%) and that of men was 443/1428 (31%). The MINORS scale showed acceptable quality in all the included studies.

### 3.3 Outcomes-meta-analysis

**Figure 2:**
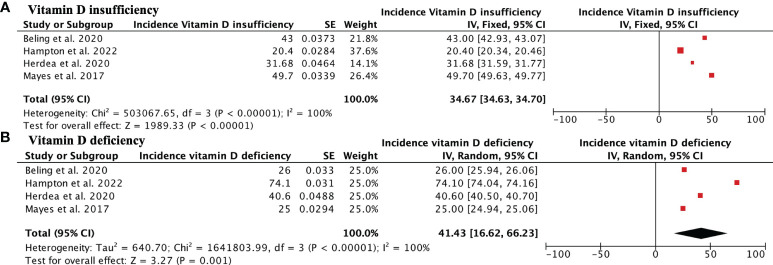
Forest plot showing the incidence of AIS in patients with vitamin D insufficiency **(A)** and deficiency. Forest plot showing significantly greater vitamin D deficiency in patients with AIS than in the control group.

**Figure 3:**
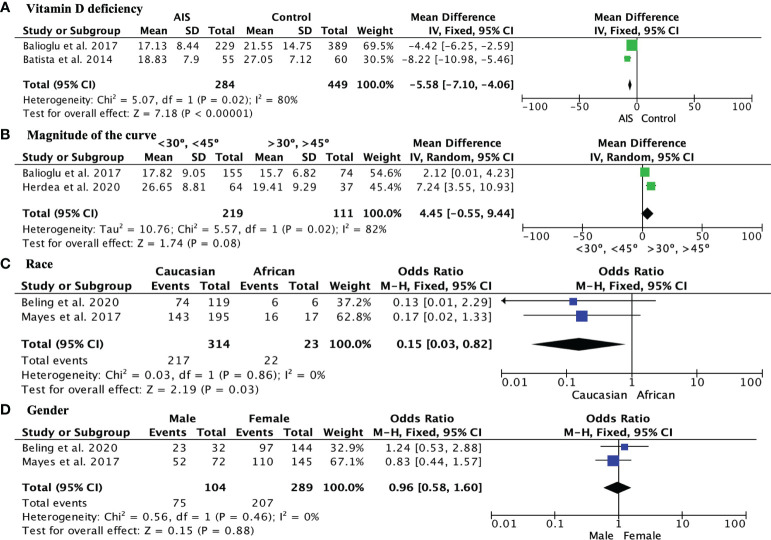
**(A)** Forest plot showing significantly greater vitamin D deficiency in AIS patients than in the control group; **(B)** Forest plot showing vitamin D levels in AIS patients with curves <30° to <45° and curves >30° to >45°; **(C)** Forest plot showing the comparison of vitamin D levels in Caucasian and African American AIS patients; **(D)** Forest plot showing the comparison of the incidence of vitamin D deficiency among AIS patients between sexes.

The incidence of vitamin D insufficiency (levels–20-29 ng/mL) in AIS patients was 36.19% (95% CI, 21.93%–50.46%), while vitamin D deficiency (<20 ng/mL) in AIS patients was 41.43% (95% CI 16.62%–66.23% (**Figure 2**). Regarding vitamin D deficiency, AIS patients had significantly lower vitamin D levels than the control group, with a mean difference of -5.58, 95% CI [-7.10, -4.06] (**Figure 3A**). When analyzing the magnitude of the curve in AIS patients, two groups were established, the first group with an angulation of “<30°-<45°” and the second group with an angulation of “>30°->45°”. No significant differences were observed in the magnitude of the deformity evaluated using the Cobb angle (MD 4.45, 95% CI -0.55 to 9.44) (**Figure 3B**). Regarding race, Caucasian patients showed a lower incidence of vitamin D deficiency than African American patients (OR 0.15, 95% CI 0.03 to 0.82; p = 0.03) (**Figure 3C**). Finally, no differences were observed in the influence of sex on vitamin D deficiency among AIS patients (OR 0.96, 95% CI 0.58 to 1.60) (**Figure 3D**).

### 3.4 Outcomes-qualitative analysis

Additionally, the selected studies assessed other risk factors or variables related to AIS that were not compared in this meta-analysis. Among the results provided, the positive correlation between vitamin D and calcium observed by Herdea et al. and Balioglu et al. (5, 14) stands out; the correlation between vitamin D deficiency and presence of pain observed by Hampton et al. (15) not evidenced by Beling et al. (17). Finally, it is interesting to note how Mayers et al. also reported a higher incidence of hypervitaminosis D in these patients in winter than in the rest of the seasons and that neuromuscular scoliosis presents with higher levels of vitamin D than idiopathic scoliosis (16).

### 3.5 Additional analyses

**Figure 4:**
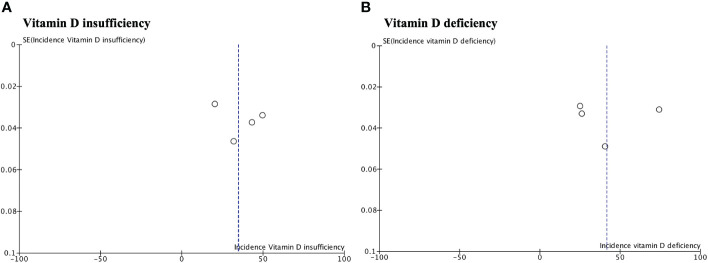
Funnel plot assessing publication bias for the incidence of vitamin D insufficiency **(A)** and deficiency **(B)**. The plot shows the distribution of the included studies according to their effect sizes. Asymmetry in the plot may appear as gaps or asymmetrical patterns, suggesting the potential omission of smaller studies with non-significant or negative results.

Publication bias was observed in the effect sizes for the incidence of vitamin D deficiency vitamin D deficiency according to the magnitude of the curve due to asymmetry (**Figure 4**).

## 4 Discussion

For this meta-analysis, six studies were selected that had the measurement of vitamin D levels in patients with AIS in common, with the aim of determining the incidence of vitamin D deficiency in this type of patient. The incidence was 36.19% in patients with vitamin D insufficiency and 41.13% in those with vitamin D deficiency. In addition, various risk factors such as sex, curve magnitude, and sex were selected to analyze their influence.

In our study, we observed a notable disparity between vitamin D insufficiency and deficiency rates among patients with Adolescent Idiopathic Scoliosis (AIS). The term “insufficiency” refers to a state where the levels of vitamin D are below the optimal range but still within a tolerable limit, while “deficiency” indicates a more severe condition where the levels fall significantly below the recommended range. The wide difference between the rates of insufficiency (35%) and deficiency (41%) suggests that a substantial proportion of AIS patients have critically low levels of vitamin D, which may have implications for skeletal health and overall well-being.

The most studied risk factor in the scientific literature is the magnitude of the curve or the Cobb angle. It has been observed that there may be a negative correlation between vitamin D levels. Several authors such as Herdea et al. (14), Ng et al. (7), and Balioglu et al. (5) studied various factors that could be affected by the lack of vitamin D in patients with AIS, and one of the most affected is the Cobb angle, which tends to progress and worsen as patients have lower levels of vitamin D or fail to normalize it. The main theory postulated to explain the influence of these two elements is that vitamin D plays a fundamental role in the process of postural balance, and it correlates positively with hip BMD and negatively with Cobb angle. Other authors, such as Zhang et al. (19), identified the Cobb angle as a triggering factor in disease progression, especially in adolescent females. A study by Sitoula et al. (20) that analyzed the correlation between Sanders Skeletal Maturity and Cobb angle concluded that both elements are predictors of curve progression in patients with AIS. Skeletal maturity and growth potential, along with the year of menarche, are factors that influence curve progression, elements that are taken into account in the study by Batista et al. (6); therefore, continuous monitoring of their pathology and vitamin D levels would be important for these patients. However, despite all these results identifying the Cobb angle as a negative factor in disease progression, the conclusion reached in the meta-analysis of this study is that the Cobb angle does not present a statistically significant relationship with vitamin D levels. These results are in line with those obtained by Hampton et al. and Beling et al., who also did not observe any association (15, 17). Therefore, we believe that further investigation is necessary to elucidate the association between the Cobb angle and vitamin D levels.

In contrast, our study also examined the influence of sex on vitamin D levels and the results of this meta-analysis showed no significant impact. The study conducted by Mayers et al. and the research carried out by Balioglu et al. (5) yielded similar results, indicating that the association between sex and vitamin D levels was not statistically significant. However, the findings of the study by Herdea et al. (14) diverged from the aforementioned results, suggesting that men with AIS have lower levels of vitamin D than women with AIS. Limited scientific literature exists regarding this risk factor, including sex and vitamin D levels, with only one consistent finding supported by strong scientific evidence: complications and exacerbating factors of AIS are more prevalent in females. According to Batista et al. (6), adolescent females affected by this condition typically exhibit taller and thinner physiques, potentially indicating abnormal growth patterns associated with vitamin D deficiency and sex. Nonetheless, further research is necessary to draw definitive conclusions on this subject.

Additionally, race was a risk factor that was found to be related to vitamin D deficiency in patients with AIS. The results obtained in the meta-analysis showed that African Americans with AIS were at a higher risk of vitamin D deficiency than Caucasians were. The same conclusion was reached in other studies, such as that of Kiebzak et al. (21). It is also worth noting that this fact may be linked to the economic resources of the population, as Beling et al. established that vitamin D deficiency is precisely associated with people with the disease who have indicators of a lower socioeconomic status (17). In any case, this risk factor has been poorly studied, as there are few studies on the subject; however, the results are statistically significant and have a correlation.

Compared to a recently published meta-analysis, this study highlights the need for further research to validate the weak correlation found between serum vitamin D levels and AIS (22). Additionally, our study provides valuable insights by reporting the specific incidence rates of vitamin D insufficiency and deficiency in patients with AIS. It also explores the relationship between vitamin D deficiency and other important factors such as patient race, curve magnitude, and sex. This helps address gaps in the understanding of the role of vitamin D in AIS, as identified in a previous meta-analysis. Further research is needed to substantiate these preliminary findings and their clinical implications.

This study has several limitations. First, differences in the study design can contribute to contradictory results, such as variations in the inclusion criteria, sample size, and follow-up period, which can affect the statistical power and generalizability of the findings. Second, the demographic data of the patients, such as age, sex, race, and comorbidities, may also influence the incidence. Additionally, the small number of articles limited the subgroup and sensitivity analyses, which, in turn, limited the power of our meta-analysis. Third, the methods used to evaluate vitamin D deficiency and different levels of vitamin D can vary. The lack of a clear definition of vitamin D deficiency can also contribute to the variability of the results, as well as the origin of the patients, which was only specified in the studies by Herdea et al. and Hampton et al. (14, 15). The contradictory results in the literature highlight the need for further research in this area with the aim of addressing the methodological limitations of previous studies and using standardized definitions and assessment tools to improve the comparability of results. Furthermore, the study design, observer experience, funding, conflicts of interest, and diagnostic criteria can influence the results of meta-analyses of vitamin D deficiency in patients with AIS and should be considered in future research. However, it may be interesting to delve into variables that were not the objective of our study, such as the possible link between the presence of vitamin D deficiency in AIS and back pain, as the only two articles that address this issue are those by Hampton et al. and Beling et al. (15, 17). Finally, it should be noted that various errors were found in the studies analyzed, such as incorrect labeling of study groups. This makes it difficult to interpret the results. Finally, regarding the magnitude of the curve, to compare the two studies, different criteria for curve magnitude were included.

## 5 Conclusion

In conclusion, this study aimed to synthesize the results obtained from different studies through literature search and meta-analysis. The analysis revealed that the incidence of vitamin D insufficiency in patients with adolescent idiopathic scoliosis was 36.19%, whereas the incidence of vitamin D deficiency was 41.43%. Sex was not a significant factor influencing vitamin D levels in patients with adolescent idiopathic scoliosis. However, race was found to be a significant factor that influences vitamin D levels, as African patients with adolescent idiopathic scoliosis were found to be at a higher risk of having low levels of vitamin D compared to Caucasians. The Cobb angle could not have exact values due to the variability in the cutoff points between the studies and did not have a significant influence on vitamin D levels in patients with adolescent idiopathic scoliosis. Additionally, there was a difference in vitamin D levels between patients with adolescent idiopathic scoliosis and the control group, as those who suffer from this condition are at a higher risk of having lower vitamin D. Finally, a sensitivity analysis could not be performed because of the small number of studies.

## Data availability statement

The original contributions presented in the study are included in the article/**Supplementary Material**. Further inquiries can be directed to the corresponding author.

## Author contributions

Conception and design: CI, GM, JO, CB. Analysis and interpretation of the data: CI, GM, JO, CB. Drafting of the article: CI, GM, CB. Critical revision of the article for important intellectual content: CB. Final approval of the article: CI, GM, JO, CB. Statistical expertise: CI, GM, JO, CB. Collection and assembly of data: CI, GM, JO, CB. All authors contributed to the article and approved the submitted version.
