# Supplementary material for: Incidence of vitamin D deficiency in adolescent idiopathic scoliosis: a meta-analysis
Source: Front Endocrinol (Lausanne). 2023 Oct 11;14:1250118. doi: 10.3389/fendo.2023.1250118 (PMC10598863; doi:10.3389/fendo.2023.1250118)
Supplement: Supplementary file 1 [file DataSheet_1.pdf]

Supplementary File 1. Pubmed search strategy.

"Vitamin D"[All Fields] AND ("scoliosis"[MeSH Terms] OR "scoliosis"[All Fields] OR "scolioses"[All Fields])

Translations

Scoliosis: "scoliosis"[MeSH Terms] OR "scoliosis"[All Fields] OR "scolioses"[All Fields]
